# Concentrations of per- and polyfluoroalkyl substances (PFAS) in paired tap water and blood samples during pregnancy

**DOI:** 10.1038/s41370-023-00581-7

**Published:** 2023-09-25

**Authors:** Dora Cserbik, Maribel Casas, Cintia Flores, Alexandra Paraian, Line Småstuen Haug, Ioar Rivas, Mariona Bustamante, Payam Dadvand, Jordi Sunyer, Martine Vrijheid, Cristina M. Villanueva

**Affiliations:** 1 ISGlobal, Barcelona, Spain; 2 Universitat Pompeu Fabra (UPF), Barcelona, Spain; 3 CIBER Epidemiología y Salud Pública (CIBERESP), Madrid, Spain; 4 Mass Spectrometry Laboratory/Organic Pollutants, Institute of Environmental Assessment and Water Research, IDAEA-CSIC Barcelona, Spain; 5 Centre for Sustainable Diets, Norwegian Institute of Public Health, Oslo, Norway; 6 IMIM (Hospital del Mar Medical Research Institute), Barcelona, Spain

**Keywords:** Drinking water, PFAS, Biomonitoring, Exposure assessment

## Abstract

**Abstract:**

Per- and polyfluoroalkyl substances (PFAS) are water-soluble chemicals of concern due to their persistence, ubiquity, and toxicity. We explored correlations between drinking water and blood PFAS levels in a subset of the mother-child Barcelona Life Study Cohort (BiSC), Barcelona, Spain (2021). For 105 study participants, we analyzed 35 PFAS in tap water (unfiltered and filtered) and 23 PFAS in 98 paired plasma samples during the 3rd trimester, using LC-MS/MS. Water consumption habits were ascertained at the third trimester through questionnaires. The majority of participants consumed bottled water (56.2%), 5/35 PFAS were detected in unfiltered tap water, 4/35 PFAS in activated carbon filtered tap water samples, and 14/23 PFAS in plasma samples. Our results showed that PFHpA at the observed concentrations in drinking water was significantly correlated with paired plasma levels (*R* = 0.2; *p* = 0.04).

**Impact statement:**

- Exposure to PFAS is an emerging public health concern. Our manuscript contributes meaningful information from a subset of the mother-child Barcelona Life Study Cohort (BiSC), reporting levels of a wide range of PFAS in paired tap water and plasma samples from a sensitive subpopulation residing away from point source contamination. Our findings draw attention to low-exposure ranges of PFAS in drinking water, and a weak but significant water-plasma correlation for PFHpA (a PFOA homologue), suggesting that drinking water can be a contributor to human exposure to PFHpA.

## Introduction

Per- and polyfluoroalkyl substances (PFAS) make up a diverse group of persistent synthetic chemicals with extensive production since the 1950 s given their water-, heat-, grease-, and oil-resistant properties [1]. Exposure to PFAS is an emerging public health concern due to their ubiquitous presence, persistence in different environmental media and human biological systems [1–3]. Some legacy PFAS such as perfluorooctanoic acid (PFOA) and perfluorooctanesulfonic acid (PFOS) and other long-chain perfluoroalkyl acids (PFAAs) have already been regulated or restricted, while shorter-chain PFAS have been introduced by the industry as replacement compounds [4]. Although PFOA and PFOS have longer half-lives and more bioaccumulative potential in humans than their alternatives, human exposure levels and potential risks are yet to be characterized for replacement and emerging PFAS [5].

Epidemiological studies have shown that exposure to PFAS, in particular PFAAs, has been associated with a range of adverse health effects such as developmental [6], immune [7], reproductive [8], hepatic [9], and metabolic disorders [10]. Specifically, exposure to PFAS have been associated with adverse pregnancy outcomes and *in-utero* exposure has been associated with developmental outcomes such as fetal and childhood growth restriction, spontaneous abortion and impacts on gestational duration [6]. In this context, prenatal exposure to PFAS may be an important driver of early-life health outcomes and predisposition to illness later in life according to the Developmental Origins of Health and Disease hypothesis. PFAS have been commonly detected in blood samples of pregnant women worldwide [11–16]. Importantly, PFAS have been shown to accumulate in the placenta and to be transferred through the placental barrier resulting in fetal exposure and potential adverse health outcomes [17].

Humans can be exposed to PFAS through multiple pathways directly or indirectly. The relative contribution of each pathway depends on the frequency of exposure, the concentration in the exposure media, and the uptake fraction [18, 19]. Dominant sources of exposure to PFAS are through drinking water, food and air or dust, while the highest environmental concentrations are observed near contaminated sites [20–22]. PFAS are found to be difficult to remove during water treatment, thus drinking water is considered the main source of human exposure to legacy and emerging PFAS near contaminated areas that is verified by biomonitoring studies [23–26]. Relative source contribution of tap water to matching serum legacy PFAS concentrations has been reported to be 20% in the U.S. [27], and 23% for PFOA in China [28], for the general population, respectively. However, information on residential exposure estimates in tap water matching blood levels is limited to a few PFAS [27]. Moreover, there is a lack of information regarding European populations residing away from point sources and their measured PFAS levels concurrent in drinking water and blood.

The aim of the present study was to explore the correlations between drinking water and blood PFAS levels in a subset of the mother-child Barcelona Life Study Cohort (BiSC) from Spain.

## Materials and methods

### Study population and sample collection

This study was nested in the BiSC, a longitudinal population-based birth cohort study in the Barcelona metropolitan area where 1080 pregnant women were enrolled between 2018 and 2021 from three main university hospitals in Barcelona (www.projectebisc.org). Information about lifestyle, home characteristics, water use and water consumption were collected through questionnaires in the first and third trimester. Questions on water consumption habits were self-administered by the participants and included the type of drinking water and amount of water consumed at home (glasses/day), the type of filter used, the type of bottled water consumed, and the type of water used for cooking.

We selected a subset of 105 BiSC participants for residential tap water sampling. Specifically, eligibility criteria for inclusion in this study were: (a) the participant to be in the 3rd trimester of pregnancy; (b) availability to collect tap water from participant’s residence in the 3rd trimester; (c) availability of plasma samples in the 3rd trimester of pregnancy. The BiSC study has been approved by the PS Mar Ethics Committee (CEIm 2018/8050/I.). All participants completed a written consent before participating in the study.

### Tap water and blood sample collection and preparation

Tap water from the participant’s home and plasma samples were collected concurrently between February and April of 2021 through home and hospital visits, respectively, at the third trimester of pregnancy.

The following methodology was considered for the drinking water sampling at residential locations of study participants: tap (unfiltered) water was collected when either unfiltered tap or bottled water were the main source of drinking water (*N* = 81 samples); filtered water was collected when it was the main type of drinking water consumed at home (*N* = 14 activated carbon (AC) filtered, *N* = 10 reverse osmosis (RO) filtered). Bottled water samples were not collected, because a previous study from the same area showed that PFAS were below detection limits in bottled water [29]. Water samples were collected by BiSC fieldworkers using sterile polypropylene bottles (250 mL) that were transported to the research center in a portable cooler with ice packs and stored at 4 °C until shipment to the laboratory for the PFAS analysis.

Blood samples (*n* = 98) corresponding to participants involved in the water sampling were collected by trained personnel using 15 mL BD Vacutainer® collection tubes (4 mL silica plastic vacutainer for serum, 5 mL silica glass vacutainer for serum, 6 mL EDTA tube for whole blood, plasma, buffy coat, and red cells). The blood samples were kept at 4 °C until processing. The EDTA tube was centrifuged at 2000 g for 10 min and plasma was transferred to a 15 mL tube which was centrifuged again at 2000 g for 10 min. Finally, plasma was aliquoted (4 × 0.5 aliquots) and stored at −80 °C until delivery for PFAS analysis.

### Laboratory analysis of PFAS in tap water and blood

Tap water samples were pre-concentrated by on-line solid phase extraction (SPE) followed by tandem mass spectrometry coupled to liquid chromatography for the analysis of 35 PFAS (10 perfluoroalkyl carboxylates [C4-C13], 10 perfluoroalkyl sulfonates [C4-C13], 3 perfluorooctane sulfonamides [PFOSA, N-MeFOSA, N-EtFOSA], 4 fluorotelomer sulfonates [FTS n:2, *n* = 4, 6, 8 and 10] and 8 ether-PFAS, including HFPO-DA (GenX), ADONA and chlorinated PFAS) at the Institute of Environmental Assessment and Water Research (IDAEA-CSIC; Barcelona, Spain). Labeled internal standards were added prior to analysis. For all LC–MS/MS analyses, a TSQ quantum triple quadrupole mass spectrometer equipped with an electrospray ionisation (ESI) source (Thermo Fisher Scientific, San Jose, CA, USA) was used. The limit of quantification (LOQ) was considered the first level of the calibration curve [29].

Blood plasma samples were analyzed for 23 PFAS (11 perfluoroalkyl carboxylates [C4-C14], 5 perfluoroalkyl sulfonates [C4-C10], 3 perfluorooctane sulfonamides [PFOSA, N-MeFOSA, N-EtFOSA], and 4 ether-PFAS [HFPO-DA (GenX), ADONA, 6:2 Cl-PFESA, 8:2 Cl-PFESA] using online solid phase extraction with ultra-high-performance LC coupled with tandem mass spectrometry at the Department of Food Safety at the Norwegian Institute of Public Health (NIPH; Oslo, Norway) [30]. The LOQ was determined as the response corresponding to a signal-to-noise ratio of 10:1 in spiked calf serum. The limit of detection (LOD) was LOQ/3 [30].

Two separate laboratories conducted PFAS instrumental analysis in plasma and tap water, where each lab is specialized in the respective matrix analyzed ensuring the highest quality data. A detailed description of the analytical methods, the quality assurance and quality control of PFAS analysis in tap water [29] and in plasma [30] has been published elsewhere.

### Statistical analysis

Descriptive statistics of PFAS in tap water and plasma were based on the samples with concentrations >LOD/LOQ.

Spearman’s rank correlation coefficients were calculated to examine correlations between PFAS concentrations in paired drinking water and plasma samples. Concentrations <LOD/LOQ were assigned LOD/2 or LOQ/2, respectively. Water concentration was assigned LOQ/2 for consumers of RO filtered or bottled water, and we assigned PFAS concentrations measured in unfiltered tap water divided by 2 for participants who consumed unfiltered and bottled water (50–50%).

Mann–Whitney *U* test was used to assess differences in plasma concentrations of PFAS in relation to tap and bottled water consumption. Analyses were carried out using R software (version 4.1.1) and statistical significance was regarded *p* < 0.05 [31].

## Results

### Study population

Characteristics of the study population are presented in Table 1. A total of 105 women participated in this study, with mean age at enrollment of 33.8 years (standard deviation (SD) = 4.9 years). The majority of participants were nulliparous (51.4%), non-smokers (78.1%), and had at least a university degree (60%). Results showed that the self-reported type of drinking water at home was bottled (56.2%), filtered tap (21.9%), unfiltered tap (12.4%), and both tap and bottled (9.5%) water; and the majority of participants reported cooking with unfiltered tap water (63.8%).

**Table 1:** Characteristics of the study population in a subset of the Barcelona Life Study Cohort (BiSC) (*n* total = 105).

Maternal characteristics	*N* = 105
	Mean (SD)
Age at enrollment (years)	33.8 (4.9)
Body mass index (kg/m^2^)^a^ (1st trimester)	24.3 (4.5)
	*N* (%)
Parity	
Nulliparous	54 (51.4%)
Multiparous	51 (48.6%)
Ethnicity	
Caucasian	76 (72.4%)
Latin American	26 (24.8%)
Asian	2 (1.9%)
Other	1 (1.0%)
Education	
≤Primary school	5 (4.8%)
Secondary or professional formation	37 (35.2%)
≥University	63 (60.0%)
Smoking^b^ (3rd trimester)	
Yes	7 (6.7%)
No	82 (78.1%)
Water consumed at home (3rd trimester)	
Tap (unfiltered)	13 (12.4%)
Filtered tap	23 (21.9%)
Tap (unfiltered) and bottled	10 (9.5%)
Bottled	59 (56.2%)
Water used for cooking^c^ (3rd trimester)	
Tap (unfiltered)	67 (63.8%)
Filtered tap	14 (13.3%)
Bottled	6 (5.7%)
Tap (unfiltered) and bottled	9 (8.6%)
Tap (unfiltered and filtered)	6 (5.7%)

^a^6 missing values in body mass index.

^b^16 missing values in smoking.

^c^3 missing values in water used for cooking.

### Occurrence of PFAS in paired tap water and plasma samples

**Fig. 1:**
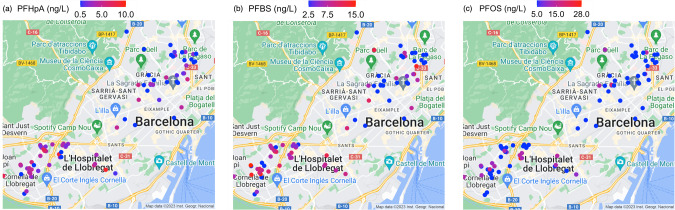
Spatial distribution of PFAS concentrations in unfiltered tap water in a subset of the Barcelona Life Study Cohort (BiSC) (*n* = 81). Plots represent mapped concentrations (ng/L) of PFHpA (**a**), PFBS (**b**), and PFOS (**c**) in residential unfiltered tap water samples (only for PFAS that were detected in paired blood samples).

Detection rates and descriptive statistics of PFAS concentrations measured in tap water and plasma samples are presented in Table 2. Among 35 target PFAS measured in tap water, five were detected in unfiltered tap water samples above the quantification limits, namely perfluoropentanoate (PFPeA; in 76.5% of samples, median = 5.8 ng/L), perfluoroheptanoate (PFHpA; 65.4%, median = 3.4 ng/L), perfluorobutane sulfonate (PFBS; 58%, median = 8.3 ng/L), PFOS (25.9%, median = 13.0 ng/L), perfluorohexanoate (PFHxA; 7.4%, median = 12.0 ng/L). Additionally, 4 PFAS were detected in AC filtered samples with lower detection frequencies and concentrations: PFPeA (71.4%, median = 4.7 ng/L), PFHpA (57.1%, median = 3.0 ng/L), PFOS (21.4%, median = 12.0 ng/L), except for PFBS which was detected in 28.6% of the samples with a median concentration of 10.5 ng/L, which was higher than that for unfiltered tap water samples. Spatial distribution of unfiltered tap water concentrations for PFHpA, PFBS, and PFOS (occurring both in water and plasma samples) revealed relatively higher concentrations in the South of the study area (Fig. 1). We did not detect PFAS in RO filtered tap water samples (Table 2). PFBA, PFCAs (C8-C13), PFSAs (C5, 6, 9–13), FTSs, PFOSAs and emerging ether-PFAS such as HFPO-DA (Gen X) and ADONA were not detected in water samples.

**Table 2:** PFAS concentrations in paired tap water and plasma samples in a subset of the Barcelona Life Study Cohort (BiSC) (*n* total = 105).

		Unfiltered tap water (*n* = 81)		Filtered tap water with AC (*n* = 14)		Filtered tap water with RO (*n* = 10)	Plasma (*n* = 98)		
	LOQ (ng/L)	>LOQ *n* (%)	Median [min, max] (ng/L)^a^	>LOQ *n* (%)	Median [min, max] (ng/L)^a^	>LOQ *n* (%)	LOD (ng/mL)	>LOD *n* (%)	Median [min, max] (ng/mL)^a^
Perfluoroalkyl carboxylates									
PFPeA (C5)	1.0	62 (76.5)	5.8 [1.4, 23.0]	10 (71.4)	4.7 [2.2, 8.2]	0	0.02	0	<LOD
PFHxA (C6)	10	6 (7.4)	12.0 [10.0, 16.0]	0	<LOQ	0	0.02	0	<LOD
PFHpA (C7)	1.0	53 (65.4)	3.4 [1.0, 9.10]	8 (57.1)	3.0 [1.0, 6.1]	0	0.02	51 (52)	0.03 [0.02, 0.1]
PFOA (C8)	10	0	<LOQ	0	<LOQ	0	0.02	98 (100)	0.6 [0.2, 1.8]
PFNA (C9)	5.0	0	<LOQ	0	<LOQ	0	0.02	97 (99)	0.2 [0.05, 0.6]
PFDA (C10)	5.0	0	<LOQ	0	<LOQ	0	0.02	97 (99)	0.1 [0.02, 0.4]
PFUnDA (C11)	5.0	0	<LOQ	0	<LOQ	0	0.02	94 (95.9)	0.2 [0.02, 0.5]
PFDoDA (C12)	10	0	<LOQ	0	<LOQ	0	0.02	80 (81.6)	0.03 [0.02, 0.1]
PFTrDA (C13)	50	0	<LOQ	0	<LOQ	0	0.02	51 (52)	0.03 [0.02, 0.2]
PFTeDA (C14)	NA	0	NA	NA	NA	NA	0.07	1 (1.0)	0.07
Perfluoroalkyl sulfonates									
PFBS (C4)	5.0	47 (58)	8.3 [4.5, 15.0]	4 (28.6)	10.5 [5.9, 15.0]	0	0.02	79 (80.6)	0.1 [0.03, 1.9]
PFHxS (C6)	10	0	<LOQ	0	<LOQ	0	0.02	98 (100)	0.2 [0.06, 1.3]
PFHpS (C7)	5.0	0	<LOQ	0	<LOQ	0	0.02	55 (56.1)	0.04 [0.02, 0.09]
PFOS (C8)	10	21 (25.9)	13.0 [10.0, 28.0]	3 (21.4)	12.0 [11.0, 21.0]	0	0.02	98 (100)	1.7 [0.3, 5.9]
Perfluorosulfonamides									
PFOSA	50	0	<LOQ	0	<LOQ	0	0.02	46 (46.9)	0.02 [0.02, 0.04]
Ether-PFAS									
6:2 Cl-PFESA	NA	NA	NA	NA	NA	NA	0.003	85 (86.7)	0.02 [0.004, 0.2]

*LOD* limit of detection, *LOQ* limit of quantification.

^a^Median values were calculated from samples >LOD/LOQ only.

NA denotes that the compound was not analyzed for the samples.

We detected 14 out of 23 target PFAS in plasma samples, of which the most frequently detected compounds (>98%, >LOD) with highest concentrations were PFOA (100%, median = 0.6 ng/mL), perfluorononanoate (PFNA; 99%, median = 0.2 ng/mL), perfluorodecanoate (PFDA; 99%, median = 0.1 ng/mL), perfluorohexanesulfonate (PFHxS; 100%, median = 0.2 ng/mL) and PFOS (100%; median = 1.7 ng/mL) (Table 2).

**Fig. 2:**
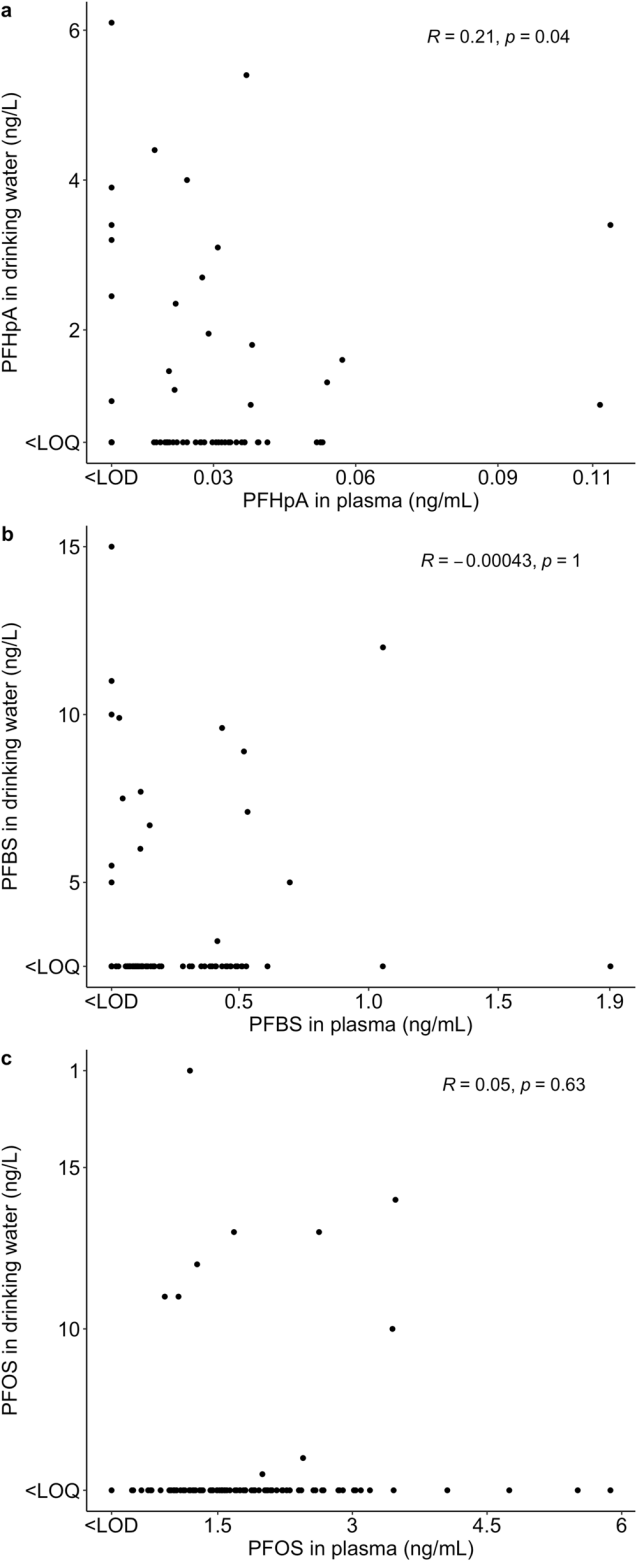
Spearman correlations of PFAS between paired drinking water and plasma samples in a subset of the Barcelona Life Study Cohort (BiSC) (*n* = 98). Spearman correlations are specific to compounds detected in paired drinking water (ng/L) and plasma samples (ng/mL): PFHpA (**a**), PFBS (**b**) and PFOS (**c**). Concentrations <LOD/LOQ were assigned LOD/2 or LOQ/2, respectively. Water concentration was assigned LOQ/2 for consumers of RO filtered or bottled water, and the measured concentration in unfiltered tap water was divided by 2 for participants who consumed both unfiltered and bottled water.

PFHpA, PFBS and PFOS were detected in both matrices (tap water and plasma, *n* = 98). We observed a weak positive correlation between plasma and water concentrations for PFHpA (Spearman correlation coefficients (*R*) = 0.21; *p* = 0.04) (Fig. 2), however, correlations were not significant for PFBS and PFOS (Spearman correlation coefficients (*R*) = −0.0004; 0.05; *p* > 0.05) (Fig. 2).

It was not feasible to estimate the ingested PFAS levels from drinking water due to the proportion of missing data (21%) on self-reported water volume intake. We did not find significant differences in PFHpA, PFBS, and PFOS plasma concentrations between participants who consumed unfiltered tap water versus bottled water.

## Discussion

To the best of our knowledge, this is the first study in Europe to report PFAS in paired blood and tap water samples in a sensitive subpopulation not residing near contaminated sites.

Results show a significant positive weak correlation between concentrations of PFHpA in paired drinking water and plasma samples among pregnant women suggesting that drinking water can contribute to PFHpA exposure even at the low-exposure range (Fig. 2). PFHpA (C7) has an intermediate carbon chain length between the long-chain perfluorinated carboxylic acids (i.e., containing ≥C7, seven perfluorinated carbons) and the short-chain perfluorinated carboxylic acids (i.e., ≤C7) [32, 33]. As such, PFHpA is structurally similar to PFOA and having the ability to readily dissolve in water as well as potentially self-aggregate in aqueous solutions while being highly resistant to degradation [33]. PFHpA detection frequencies of 24–52% in tap water has been previously reported in Barcelona [29], which is lower than in this study (65.4%). As PFHpA is a PFOA homologue, bioaccumulation potential is assumed through binding to proteins in plasma and liver, as well as having a longer serum elimination half-life in humans ranging between 1.2–2.5 years [34]. PFBS and PFOS were also present in both matrices but did not exhibit significant correlations. To note, we identified a high proportion of bottled water consumers that was taken into account for the correlation analysis. Other than PFHpA, a plausible explanation for the lack of water-blood correlations for PFBS and PFOS could be that plasma concentrations are driven by other exposure routes such as dust and food intake (fish, eggs, fruits) [3]. Previous studies reported significant associations between tap water and blood concentrations of PFOA and PFNA in U.S. women (Nurses’ Health Study (NHS) nationwide prospective cohort) who consumed >8 cups of tap water per day based on samples collected in 1989–1990 [27], and for PFOA levels in a more recent study from China (2015–2017) on the general population level [28].

With respect to PFAS in drinking water, a decline in detection frequency of PFOS with steady concentrations in tap water (unfiltered) was observed in Barcelona over time, while the detection frequency and concentrations of short-chain replacement PFAS such as PFPeA and PFBS have increased [29, 35, 36]. The presence of legacy PFAS in tap water samples remains a concern due to their persistence in the environment and resulting human exposure. The study area (the Barcelona metropolitan area) is supplied by drinking water coming from two rivers (Llobregat, Ter), and desalinated sea water [37]. The drinking water supplied is a varying a mixture of these 3 sources [37]. Observed spatial distribution of PFHpA, PFBS, and PFOS showed relatively higher concentrations in unfiltered tap water for samples collected in the Southern area (Fig. 1), which received a higher proportion of Llobregat river [37]. This may be explained by the background contamination due to the industrial activity along the Llobregat watercourse as well as the proximity to the airport [38]. However, total PFAS concentrations detected in unfiltered tap water (median = 21.0 ng/L [minimum = 1.40 ng/L, maximum = 53.0 ng/L]) in this study were below the maximum PFAS contaminant levels (sum of 20 carboxylates and sulfonates = 100 ng/L; total PFAS concentrations = 500 ng/L) set by the by EU Drinking Water Directive (EU DWD 2020/2184) [39].

In our population of pregnant women, the median concentrations of PFOS, PFOA, PFHxS, PFNA, PFUnDA in blood (1.7 ng/mL, 0.6 ng/mL, 0.3 ng/mL, 0.2 ng/mL, 0.2 ng/mL, respectively) were much lower compared to pregnant women of other European cohorts (BIB, EDEN, INMA, KANC, MoBa, RHEA) of the Early-Life Exposome project (HELIX) (6.4 ng/mL, 2.3 ng/mL, 0.6 ng/mL, 0.7 ng/mL, 0.2 ng/mL, respectively) conducted between 1999–2010 [13], while concentrations were similar to findings of a more recent US study (2014–2018) (median: PFOS = 1.9 ng/mL; PFOA = 0.8 ng/mL; PFHxS=0.3 ng/mL; PFNA = 0.3 ng/mL) [40]. Comparison of PFAS levels among cohort studies is complex as the plasma samples in this study were collected in the late pregnancy period (3rd trimester). Therefore, it is possible that PFAS levels could be lower than in the 1st trimester or compared to non-pregnant women due to pregnancy hemodynamics (e.g., increased blood plasma volume) resulting in dilution of plasma PFAS concentrations [41]. Nevertheless, detected blood PFAS concentrations have been reported to be decreasing over time as some PFAS were restricted or phased out of industrial applications due to the environmental and human health concerns [42].

A major strength of this study is the wide range of PFAS analyzed in tap water and plasma samples collected at the same period of pregnancy (3^rd^ trimester) in line with the information regarding the type of water consumed, in a sensitive subpopulation. It is noteworthy that PFBS (C4), a short-chain PFAS has been dominant in this study in unfiltered-, AC filtered tap water, and in plasma samples, while it is already known that replacement PFAS, such as PFBS, can cross the placental barrier more efficiently than long-chain PFAAs [43] and thus future research is needed to determine their impact on developmental and early life outcomes. In this respect, the scope of the current study was limited, however, future research for the overall BiSC will examine and elucidate potential health effects of PFAS. There are some limitations to the present study in relation to the small sample size and missing data on self-reported drinking water consumption volume.

## Conclusions

We report levels of a wide range of PFAS in paired tap water and plasma samples for pregnant women in the third trimester of pregnancy living in Barcelona (Spain). Findings show that PFHpA at the observed concentrations in drinking water was significantly correlated with paired plasma levels, while the correlations for PFBS and PFOS were not significant. This is the first study to suggest that drinking water can be a contributor to human exposure to PFHpA even at the low-exposure range.

### Supplementary information

Reporting Checklist

## Data Availability

All the data supporting the findings of this study is available in the article.
